# Different activation in left dorsolateral prefrontal cortex and left orbitofrontal cortex during autobiographical memory tasks is associated with depressive disorder with different levels of resilience: a functional near-infrared spectroscopy study

**DOI:** 10.3389/fpsyg.2025.1495821

**Published:** 2025-09-01

**Authors:** Huifen Wu, Baoquan Lu, Nian Xiang, Min Qiu, Hui Da, Qiang Xiao, Yan Zhang, Hui Shi

**Affiliations:** ^1^Hubei Engineering University, Xiaogan, Hubei, China; ^2^Huazhong University of Science and Technology, Wuhan, Hubei, China; ^3^Beijing Chaoyang Hospital, Capital Medical University, Beijing, China

**Keywords:** depression, resilience, fNIRS, orbitofrontal cortex, dorsolateral prefrontal cortex

## Abstract

**Objective:**

Previous studies have found that resilience is a protective factor against depression, and new antidepressant methods can be developed from the perspective of resilience. However, it remains unclear how resilience protects individuals from depressive symptoms and what neural mechanisms underlie this “protective” effect.

**Methods:**

We recruited 237 participants in our study according to the depression and anxiety clinical scale (HADS) and Connor-Davidson Resilience Scale (CD-RISC), including 100 healthy controls (HADS≤7) and 137 depressed patients (HADS≥8). All participants were evaluated using 53-channel functional near-infrared spectroscopy (fNIRS) to detect cerebral hemodynamic differences during autobiographical memory tasks.

**Results:**

The results showed that (1) the activation of oxy-Hb in the left dorsolateral prefrontal cortex (DLPFC) was significantly higher in the positive emotional valence condition than in the negative emotional valence condition for the groups of depression-high resilience and healthy-low resilience, while there was no significant difference between the positive and negative emotional valences observed in response to for the groups of depression-low resilience and healthy-high resilience. (2) Oxy-Hb activation under positive emotional valence was significantly higher in the group with healthy-low resilience than healthy-high resilience and depression-low resilience. (3) Under the negative emotional valence condition, resilience mediated the indirect effect of depression on oxy-Hb activation in the left orbitofrontal cortex (OFC).

**Conclusion:**

fNIRS may be a useful tool for diagnosing and characterizing depression in patients with high or low resilience and improving individual resilience may be a new perspective for diagnosing and intervening in depression.

## 1 Introduction

Major depressive disorder (MDD) affects 2–5% of the global population and is the number one disability worldwide, which is increasing (Kessler et al., 1994; Nestler et al., 2002; Berton and Nestler, 2006; Ferrari et al., 2013), but less than half of patients achieve complete remission, and many do not respond to currently available treatments, including antidepressants and psychotherapy (Nestler et al., 2002; Berton and Nestler, 2006; Ferrari et al., 2013; Nestler and Carlezon, 2006; Toseeb et al., 2014). One possible reason for this is that we have an incomplete understanding of the heterogeneity of depression, with wide individual differences in response to chronic or severe stress, which are important risk factors for depression (Han and Nestler, 2017). The study of resilience and its neurobiological basis is a relatively new area of scientific research (Southwick and Charney, 2012). As defined by the American Psychological Association (2013), resilience is the process of adaptation to adversity, trauma, tragedy, threats, and even major sources of stress. Increasing evidence supports the view that the promoting mechanisms of natural resilience represent a bonafide alternative path for generating novel antidepressant treatments (Han and Nestler, 2017; Friedman et al., 2014; Friedman et al., 2016). Therefore, resilience may be a protective factor against depression. Many researchers have assumed that resilience is not simply the result of a lack of disease processes, but also reflects the work of biologically based active adaptation mechanisms (Friedman et al., 2014; Russo et al., 2012). Block and Kremen (1996) argued that resilience is a continuum and that individuals with high resilience are characterized by their ability to engage in appropriate and dynamic self-regulation, whereas individuals with low resilience tend to rigidly engage in self-regulation under or over self-regulate. This dynamic and appropriate ability to self-regulate allows people with high-resilience traits to adapt more quickly to changing circumstances. Studies have also found that highly resilient individuals recover more quickly (Tugade and Fredrickson, 2004) and completely (Waugh et al., 2008a; Waugh et al., 2008b) after being threatened by possible negative experiences and that highly than resilient individuals cope better with stress and report more positive emotions than resilient individuals. They are also less likely to report depressive symptoms (Klasen et al., 2015; Ong et al., 2006). In summary, it is of great theoretical and practical significance to develop new treatment and prevention methods for depression to explore the differences in brain activation between healthy individuals and patients with depression with high and low resilience and further clarify the neural mechanism of resilience as a protective effect against depression.

Although the American Psychological Association (2013) has defined resilience, scholars have provided different definitions according to their research fields. Some studies have defined resilience as a process of dynamic adaptation to adverse and unpleasant experiences (Luthar et al., 2000), but it generally refers to an individual’s ability to face stressful events (Yi‐Frazier et al., 2010). It has also been defined as a functional mode that indicates positive adaptation to a risky or adverse environment (Ong et al., 2009). Resilience refers to resistance to stress (Garmezy, 1985), corresponds to successful adaptation (Donnellan et al., 2009), and behavioral adjustment (Leve et al., 2009). Although there are many definitions of resilience, we can generally divide the study of resilience into two categories based on commonalities. One major line of research considers resilience in the context of development and argues that resilience is a dynamic process, an individual’s ability to adapt to adversity, rather than a ‘trait,’ and is distributed throughout the system of child development (Masten, 2014a; Masten, 2014b). According to this concept, resilience develops through the capabilities in the development of different systems. However, another group sees resilience as focusing on youth and adult resilience in the context of development, seeing it as a measurable trait and taking a psychopathological rather than a developmental view. According to this view, trait resilience is a personality trait that favors positive adaptation to loss and can be measured and modified (Connor and Davidson, 2003; Campbell-Sills et al., 2006; Waugh et al., 2008a; Waugh et al., 2008b). This study considered resilience from the latter perspective and used a self-report questionnaire (Connor-Davidson Resilience Scale, CD-RISC) to measure individual trait resilience.

The PFC participates in trait resilience, especially in the interaction between the orbitofrontal cortex (OFC) and dorsolateral prefrontal cortex (DLPFC) in trait resilience, which is a neurophysiological mechanism of trait resilience (Salehinejad et al., 2017). The OFC is involved in trait resilience through emotional flexibility (Waugh et al., 2008a; Waugh et al., 2008b; Lieberman et al., 2007; Phillips et al., 2008). The OFC is extensively involved in affective and cognitive integration (Rolls, 1999) and, more specifically, in the expectation of negative outcomes (O'Doherty et al., 2001). This is especially true when the outcome is more negative than the alternative (Waugh et al., 2008a; Waugh et al., 2008b). The OFC activity increases when aversive or threatening stimuli are expected. However, individuals with high resilience show reduced OFC activity in this situation compared to individuals with low resilience because they have greater emotional flexibility (Waugh et al., 2008a; Waugh et al., 2008b). Reynaud et al. (2013) also reported functional activation in the right amygdala and left orbitofrontal cortex (LOFC) in firefighters who scored high in trait tolerance (a structure associated with resilience). It is associated with cognitive flexibility, reassessment, and impulse control (Steinbeis et al., 2012). These skills are known to contribute to mental health and are impaired in major depression. Skills, such as restructuring and cognitive flexibility, have been identified as part of resilience (Iacoviello and Charney, 2014). The left DLPFC volume was significantly higher in the healthy high-risk group than in the other groups. The DLPFC is associated with cognitive and emotional processes, and high volumes of the DLPFC in this region can contribute to adaptive coping in high-risk individuals to maintain their mental health. Therefore, this increased volume may constitute the neural basis for resilience to major depression in high-risk individuals (Brosch et al., 2022). Sanchez-Lopez et al. (2020) used transcranial direct current stimulation (tDCS) of the left DLPFC to improve individual attention and emotional regulation processes (improved re-appraisal and reduced rumination) and promote improved mental resilience. In conclusion, these studies suggest that the LOFC and left DLFPC may be involved in the neurophysiological mechanisms of resilience.

fNIRS, a relatively new and promising neuroimaging method, is a psychophysiologically useful and objective indicator of cognitive function in clinical settings, which has a higher temporal resolution (0.1 s) than functional magnetic resonance imaging (fMRI) and higher spatial resolution (2–3 cm) than electroencephalograms (EEGs), and can be used relatively quickly to measure brain function dynamics while being insensitive to noise and head movements (Lai et al., 2017), and able to overcome interference of cognitive neuro-techniques (e.g., MRI) due to task-generated noise (Pinti et al., 2020). Many fNIRS studies have found that patients with depression, as opposed to controls, are associated with a reduced rise in oxy-Hb levels in the PFC during cognitive tasks (Zhang et al., 2015; Ho et al., 2020). Ho et al. (2020) further proposed that a distinct and consistent characteristic pattern of blood oxygenation changes in the prefrontal cortex in response to different cognitive tasks between patients with depression and healthy controls using fNIRS could be used as a tool to assist in depression diagnosis, predict patients’ clinical symptoms, monitor treatment response and disease progression, and predict the disease. Recent studies have used functional near-infrared spectroscopy (fNIRS) combined with autobiographical memory tasks (AMTs) to explore differences in PFC activation between healthy individuals and patients with depression (subtypes and severities of depression) from the perspective of emotion and cognition and found that differences in oxy-Hb activation under different emotional states can be used to distinguish healthy individuals from patients with depression and various subtypes and severities of depression (Zhang et al., 2022; Zheng et al., 2023; Wu et al., 2024; Zhang et al., 2025). For example, Wu et al. (2024) found that hemodynamic hyperactivation of negative emotional valence in the left DLPFC during AMT may be due to neurophysiological differences between anxious and non-anxious patients with depression. Zhang et al. (2025) found that the left OFC mediated the process from insomnia to depression, while the left DLPFC directly moderated the impact of insomnia on depression and the influence of the left OFC on depression. The authors further proposed that interventions in the left OFC and DLPFC in the insomnia population potentially alleviate depressive symptoms during AMT. Further, Zheng et al. suggested that depressed individuals with suicidal ideation have deficits in executive function in the right dlPFC, while depressed adults without suicidal ideation may have a mechanism of resource compensatory recruitment in the left dlPFC, and the dlPFC abnormality involved in the pathophysiology may localize within the left hemisphere during AMT (Zheng et al., 2023). These studies further illustrate that when using fNIRS as a tool to diagnose depression, AMT is an effective and feasible task. Therefore, this study used fNIRS and AMT to explore differences in brain activation between healthy individuals and patients with depression with high and low levels of resilience and further clarify the neural mechanism of elasticity as a protective effect against depression to provide a new perspective for the diagnosis and intervention of depression.

Previous studies suggest that the LOFC and left DLFPC may be involved in the neurophysiological mechanisms of resilience (Salehinejad et al., 2017; Waugh et al., 2008a; Waugh et al., 2008b; Lieberman et al., 2007; Phillips et al., 2008; Brosch et al., 2022; Sanchez-Lopez et al., 2020). Many fNIRS studies have confirmed that patients with depression have insufficient activation in the left DLPFC and the LOFC (Shajahan et al., 2002; Zhong et al., 2011; Mannie et al., 2011; Ong et al., 2021) and that the degree of activation in these regions are negatively correlated with the level of depression in depressed patients (Shajahan et al., 2002; Speer et al., 2009; Zhong et al., 2011). As can be seen from the above discussion, resilience and depression share the same physiological mechanisms, which provides a neurophysiological basis for finding methods of intervention and treatment for depression from the perspective of resilience. This study takes the left DLPFC and LOFC as regions of interest and compares differences in brain activation among healthy-high resilience, healthy-low resilience, depression-high resilience, and depression-low resilience to reveal how resilience might protect individuals from depressive symptoms and what neural mechanisms could underlie this “protective” effect.

Furthermore, although many studies found a significant negative correlation between depressive symptoms and oxy-Hb concentrations in the left DLPFC and LOFC using fNIRS (Shajahan et al., 2002; Zhong et al., 2011; Mannie et al., 2011; Speer et al., 2009; Ong et al., 2021), other studies have observed no significant correlations (Tsujii et al., 2014) or even a significant positive association (Liu et al., 2014) between depression scores and oxy-Hb concentrations. Some researchers believe that resilience is a protective factor against depression and that individuals with high resilience are not prone to depression. Therefore, new measures of depression can be developed from the perspective of resilience (Southwick and Charney, 2012). This study also used the left DLPFC and LOFC as regions of interest and explored whether resilience plays a mediating role in the influence of the degree of depression on hemodynamic activation, increasing our understanding of how resilience exerts a protective effect on depression, improving the accuracy and reliability of fNIRS in diagnosing depression, and developing new approaches for the intervention and treatment of depression.

## 2 Materials and methods

### 2.1 Participants

This study included 237 participants: 100 healthy controls and 137 patients with depression. Participants were recruited from the Psychiatry Department of Huazhong University of Science and Technology in Wuhan, China, from September 2019 to October 2022. The patients with depression in this study were young college students aged 18–20 years who were right-handed and were diagnosed with DSM-5 by three senior psychiatrists. Considering that the patients with depression in our study were young, we recruited healthy controls from Huazhong University of Science and Technology to better match their age, sex, and education. Before fNIRS monitoring, all participants were assessed using the Mini International Neuropsychiatric Interview (Chinese version) by senior psychiatric clinicians according to DSM-5 standards and the Hospital Anxiety and Depression Scale (HADS), which satisfied the criteria for depression.

We selected healthy controls (100) and patients with depression (137) who scored above or below 27% on the Connor-Davidson Resilience Scale (CD-RISC) in healthy controls and depressed patients. Participants with the same critical-point scores in the high- and low-resilience groups were excluded. Ultimately, 128 participants were selected: 27 healthy-high resilience, 27 healthy-low resilience, 37 depression-high resilience and 37depression-low resilience. Finally, 46 healthy controls and 54 patients with depression were excluded. The four groups were matched for sex, age, and years of education (Table 1).

**Table 1:** Demographic characteristics and clinical scale assessments of healthy controls and depression with different levels of resilience.

	Healthy-high resilience	Healthy-low resilience	Depression-high resilience	Depression-low resilience	*F/x^2^*	*p*
Age	18.700 ± 0.724	18.590 ± 0.844	18.500 ± 0.609	18.840 ± 0.638	1.189	0.189^a^
Education (years)	12.704 ± 0.724	12.592 ± 0.844	12.778 ± 0.637	13.158 ± 0.547	1.354	0.260^a^
Gender (female/male)	5/22	7/20	9/25	10/27	0.023	0.511^b^
HAD-D	2.780 ± 1.695	5.580 ± 1.099	11.500 ± 2.223	11.660 ± 2.374	134.550	*p* < 0.001^a^
CD-RISC	112.590 ± 3.630	79.520 ± 5.937	94.330 ± 12.788	57.790 ± 7.516	230.584	*p* < 0.001^a^

Values are expressed as mean ± SD [range]. The *p*-values are derived from the χ^2^ test or one-way ANOVA. ‘a’ represents one-way ANOVA; ‘b’ represents the χ^2^ test.

Exclusion criteria were as follows: left-handedness, history of other mental illnesses, history of brain trauma or other organic brain diseases, substance-related or addictive disorders, history of significant physical illness, pregnancy and lactation, received electrical shock therapy within the last 6 months, and inability to complete cognitive tasks. All participants were native Chinese speakers. This study was approved by the Ethics Committee of the Huazhong University of Science and Technology.

### 2.2 Psychological assessment

#### 2.2.1 HADS

The HADS is a 14-item self-report measure with seven items that form a depression subscale and seven items that measure anxiety (Zigmond and Snaith, 1983). Each item is rated on a four-point scale ranging from 0 to 3, with 3 indicating a higher frequency of symptoms. The total score for each subscale ranges from 0 to 21, categorized as normal depression/anxiety (0–7), suspected depression/anxiety (8–10), or diagnosed depression/anxiety (11–21). The participants read and answered questions in the presence of an interviewer. The results of the depression “D” scale were examined. For the inclusion of depressed patients, scores ≥8 on the depression rating scale were selected.

The HADS is a unidimensional scale designed as a brief self-report measure of the severity of depressive or anxious states (Snaith, 1992) and has been widely used to assess the symptom severity of anxiety disorders and depression among different populations (Bjelland et al., 2002; White et al., 1999). The reliability and validity of the scale have been verified in various languages (including Chinese) (Whelan-Goodinson et al., 2009; Herrero et al., 2003). Many fNIRS studies on depression have used the HAD scale to distinguish patients with different severities and subtypes of depression, detect differences in brain blood oxygen activation, and search for biomarkers and effective intervention methods to provide a basis for efficient diagnosis and treatment of depression (Zhang et al., 2022; Wu et al., 2022; Zheng et al., 2023).

#### 2.2.2 Connor-Davidson resilience scale (CD-RISC)

The CD-RISC in Chinese people consists of 25 items, each of which is rated on a five-point scale, ranging from 1 to 5 (Yu and Zhang, 2007). This scale measures positive psychological qualities that are conducive to the adaptation of individuals to adversity (Connor and Davidson, 2003). The higher the score, the higher the level of thought resilience. We selected participants with scores above and below 27% of the CD-RISC scores of healthy controls and depressed patients as participants, and those with the same critical-point scores of the high- and low-resilience groups were excluded.

### 2.3 Activation task (AMT)

The two pictures (positive and negative) used in this study were derived from the Chinese Affective Picture System (CAPS), a localized affective picture system in China that has been proven to have high internal consistency and test–retest reliability in valence, arousal, and dominance (Bai et al., 2005). During the AMT, the experimental task stimuli were presented to the participants using PowerPoint and the order of presentation of the two emotional pictures was randomized. Participants and the experimenter were not allowed to communicate during the AMT.

**Figure 1:**
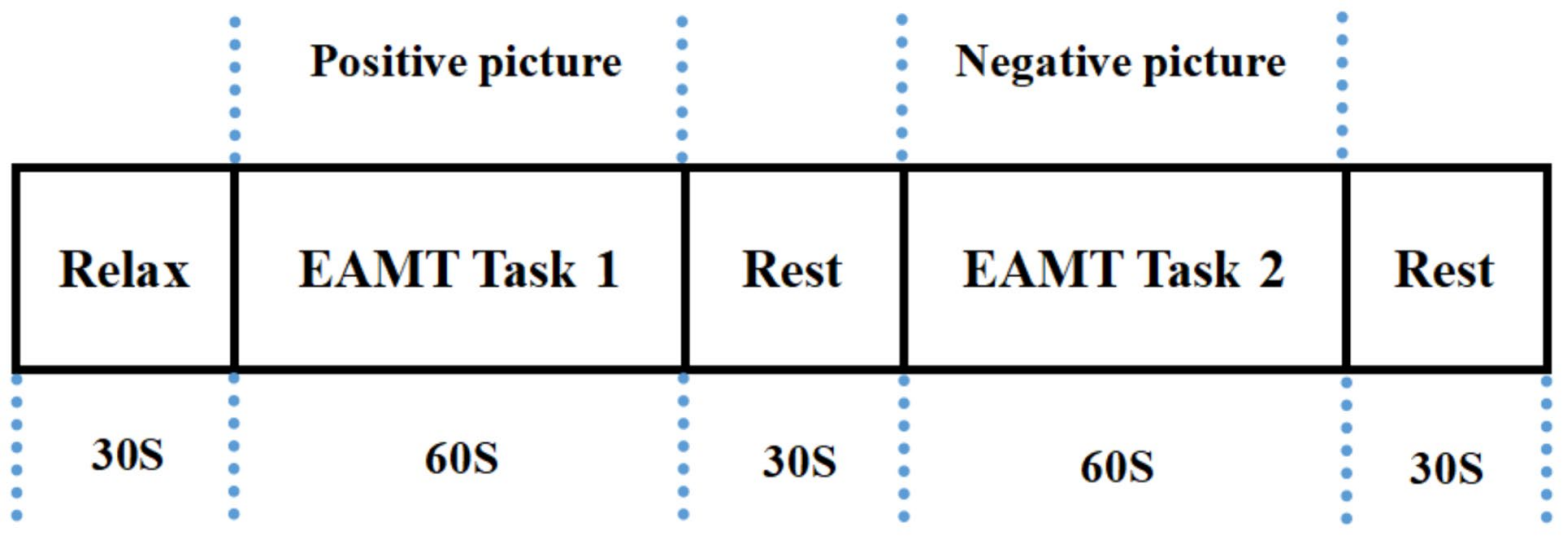
Experiment procedure.

The total duration of the AMT was approximately 210 s and contained the following three stages. Stage 1: 30s of pre-task rest. Participants continued counting from 1 to the end of Stage 1 for a total time of 30 s. Stage 2: 150 s AMT task. The participants recalled positive or negative events as much and as quickly as possible and were simultaneously asked to orally describe the recalled events (including time, situations, places, and people) as specifically and in detail as possible for 60 s according to the emotional pictures, closing their eyes for a 30-s rest between positive and negative events. Stage 3: 30s post-task rest. Participants continued to count from 1 to the end of the experiment for a total time of 30s. The task design is illustrated in Figure 1 (Wu et al., 2024).

It is worth mentioning that we did not measure the response time or number of positive and negative episode judgments. However, we assessed the accuracy of the participants’ recollections. Based on the content of the participants’ oral recollections, we excluded those who did not recall as required. As our study aimed to use fNIRS to investigate hemodynamic activation in the left DLPFC and LOFC of groups with healthy-low resilience, healthy-high resilience, depression-low resilience and depression-high resilience, we recorded the participants’ brain activation during AMT triggered by different emotional valence stimuli.

### 2.4 Near-infrared spectroscopic imaging measurement

**Figure 2:**
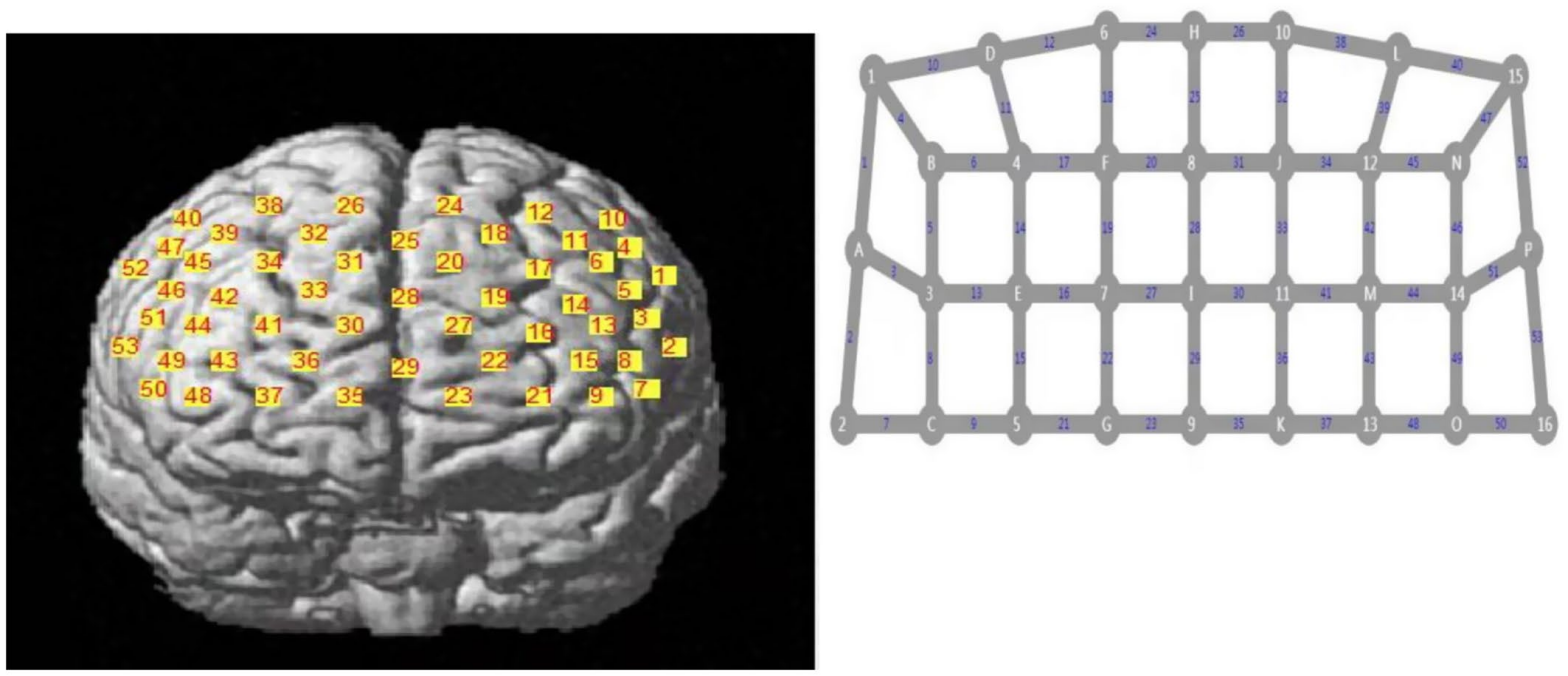
Optode arrangement.

A 53 channel fNIRS device (BS-7000, Wuhan Znion Technology Co., Ltd., China) was used to measure changes in the three types of relative concentrations of oxy-Hb, deoxy-Hb, and total-Hb using near-infrared light of a specific wavelength. The instrument contains 16 pairs of emission and detector probes. The wavelengths of the emitted light were 760 and 850 nm and the frequency was 15.625 Hz. The distance between each emitter and detector was 2.9–3.1 cm. The area between the emitter and detector probes consisted of a channel. The probes were placed on the forehead of the scalp. The optode arrangement is shown in Figure 2 (Wu et al., 2022; Wu et al., 2024) and is based on the 10/20 electrode placement method commonly used in electroencephalograms (Okamoto et al., 2004).

Sixteen pairs of optodes and 53 channels. The numbers represent the emitter probes and the letters represent the detector probes.

### 2.5 Regions of interest

The left prefrontal area, where there was a change in the oxy-Hb waveform during AMT, was of particular interest. Therefore, the regions of interest (ROIs) were set in the following regions: the left dorsolateral prefrontal cortex (DLPFC) (6th, 9th, 11th, 13th, 14th, 15th, 16th, 17th, 18th, 19th, 20th, and 25th channels) and left orbitofrontal cortex (LOFC) (21st and 23th channels) (Figure 2).

### 2.6 Statistical analysis

A one-way analysis of variance (ANOVA) was performed for the groups with healthy-low resilience, healthy-high resilience, depression-low resilience and depression-high resilience according to age and years of education. Gender frequency between groups was compared with a χ^2^ test. We performed a 2 (groups: healthy controls/depression) × 2 (resilience levels: high/low) × 2 (emotional valence: positive/negative) repeated-measures ANOVA and a simple effect analysis or simple effect analysis for oxy-Hb activation in ROIs. A post-hoc pairwise comparison analysis was conducted and the statistical significance was set at *p* < 0.05. We used the Bonferroni correction to address multiple comparisons with adjusted *p*-values for *post hoc* tests to compare two or three specific groups. SPSS 20.0 was used for all statistical analyses. Furthermore, we used SPSS INDIRECT MACRO version 3.4 (Preacher and Hayes, 2008), a multiple regression procedure, to test for the mediation effects of resilience level (mediation assessment by Model 4 in the process) on depression severity and oxy-Hb changes in specific brain regions. Subsequently, we used the bootstrapping method to test the significance of the indirect effect using 5,000 bootstrap samples and 95% confidence intervals (CIs) (Shrout and Bolger, 2002). CIs that did not include zero were considered statistically significant.

fNIRS data were analyzed using Homer2 software (Huppert et al., 2009), and the processing procedure and related parameters were those referenced in Brigadoi et al. (2014). We deleted channels in the data that were oversaturated with light intensity. The relative coefficient of variation (CV; %) was used to identify poor channels (Piper et al., 2014). Participants whose measured channels had CVchan of >15% were excluded. Although participants with CVchan of <15% were retained, bad channels were counted as empty values in the statistical analysis.

For each participant, the original light intensity data were converted into relative changes in optical density (OD). Motion artifacts in the OD data were then identified and corrected by moving the standard deviation and cubic spline interpolation (Scholkmann et al., 2010). Physiological signals were removed from the data using a low-pass filter with a cut-off of 0.2 Hz. Low-frequency drift was removed by a high-pass filter with a cut-off frequency of 0.01 Hz (Arun et al., 2020). After filtering, the OD data were converted into oxy-Hb and deoxy-Hb concentration data following the Beer–Lambert law.

Regarding fNIRS data, we focused on oxy-Hb changes because oxy-Hb has a superior signal-to-noise ratio and can reflect task-related cortical activation more directly than deoxy-Hb (Huppert et al., 2006; Strangman et al., 2002). Thus, we calculated the mean activation changes in oxy-Hb in the 53 channels by determining the difference in the mean oxy-Hb values between the task and pre-task periods.

## 3 Results

### 3.1 Demographic characteristics of the participants and clinical scale assessment

There were no significant baseline differences in education, age and gender among the groups with healthy-low resilience, healthy-high resilience, depression-low resilience and depression-high resilience (*p* > 0.05) (Table 1).

The four groups had comparable HADS scores for depression (*p* < 0.001) and RRS scores for resilience (*p* < 0.001). Compared to the groups with healthy-high resilience and depression-high resilience, the groups with healthy-low resilience (*p* < 0.001) and depression-low resilience (*p* < 0.001) showed lower CD-RISC resilience scores. Compared to the groups with healthy-high resilience and healthy-low resilience, the groups with depression-high resilience (*p* < 0.001) and depression-low resilience (*p* < 0.001) showed higher HADS depression scores (Table 1).

### 3.2 Comparison of hemodynamic response during the AM task between the groups

We performed a 2 (groups: healthy controls/depression) × 2 (resilience levels: high/low) × 3 (emotional valence: positive/negative) repeated-measures ANOVA for oxy-Hb activation in the left DLPFC. There was a significant main effect of emotional valence (ch6, ch9, ch11, ch13-20; *p* < 0.05), while there was no significant main effect of the groups (*p* > 0.05) or resilience level (*p* > 0.05). There was no significant interaction effect of groups × emotional valence (*p* > 0.05), while there were significant interaction effects of the emotional valence × resilience level (ch9; *p* < 0.05), groups × resilience level (ch19; *p* < 0.05), and groups × resilience level × emotional valence (ch15; *p* < 0.05). In view of the above, three channels (9, 15, and 19) with interactions were all located in the left DLPFC. When the three-way interaction was significant, the higher-way interaction was analyzed first (Wickens and Keppel, 2004), and there was no need to explain the significant two-way interaction and the main effect separately (Jaccard and Turrisi, 2003). Therefore, the simple effects analysis only analyzed ch15.

**Figure 3:**
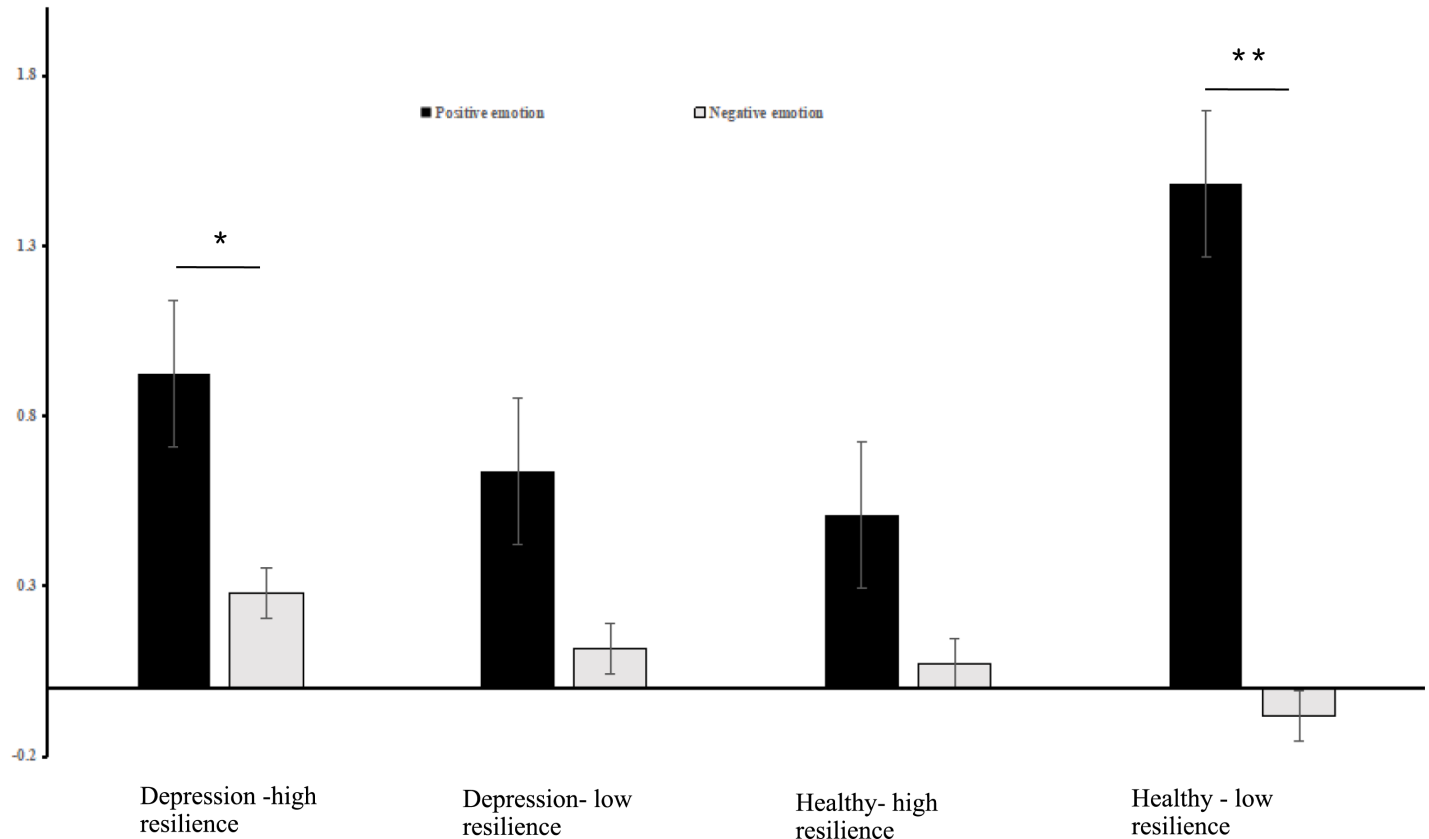
Histogram of the distribution of the average oxy-Hb activation in the left DLPFC in Ch15 under positive and negative emotional valence among the groups with healthy-low resilience, healthy-high resilience, depression-low resilience and depression-high resilience. ^*^p < 0.05, ^**^p < 0.001.

**Figure 4:**
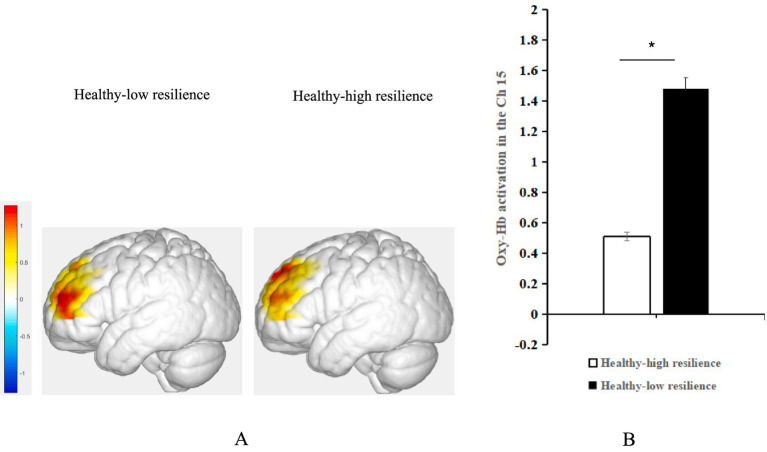
**(A)** Average activation of oxy-Hb in the left DLPFC with positive emotional valence between the groups with healthy-high resilience and healthy-low resilience during AMT. **(B)** Histogram of the distribution of average oxy-Hb activation in Ch15 under positive emotional valence between healthy control with high resilience and low resilience. *p < 0.05.

**Figure 5:**
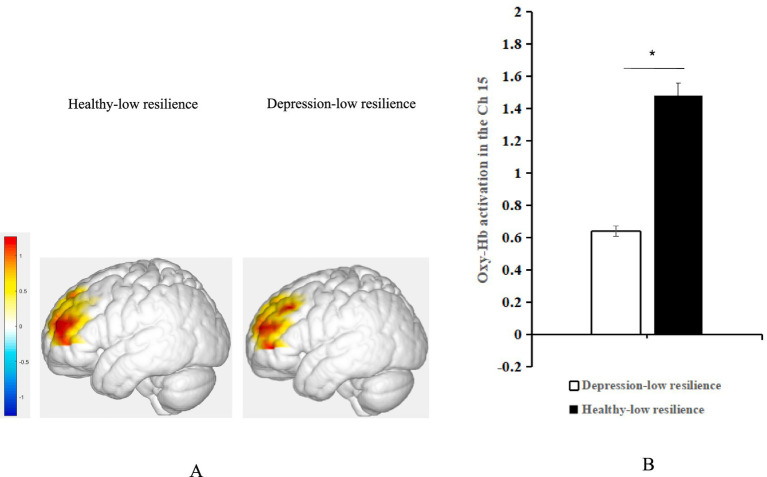
**(A)** Average oxy-Hb activation of the left DLPFC under positive emotional valence between the groups with depression-low resilience and healthy-low resilience during AMT. **(B)** Histogram of the distribution of average oxy-Hb activation in Ch15 under positive emotional valence between healthy control with low resilience and depression with low resilience. *p < 0.05.

Simple effect analysis showed that the activation of oxy-Hb in the left DLPFC was significantly higher in positive emotional valence than in negative emotional valence for the groups with depression-high resilience (Bonferroni-corrected *p* < 0.05/4) and healthy-low resilience (Bonferroni-corrected *p* < 0.05/4), whereas there were no significant differences between the two emotional valences for the groups with depression-low resilience (*p* > 0.05) and healthy-high resilience (*p* > 0.05) (Figure 3). Oxy-Hb activation in the positive emotional valence for the group with healthy-low resilience was significantly higher than with healthy-high resilience (Bonferroni-corrected *p* < 0.05/4) (Figure 4), while oxy-Hb activation in the positive emotional valence for the group with depression-low resilience was not significantly different from that of depression-high resilience (*p* > 0.05). Oxy-Hb activation in the positive emotional valence for the group with healthy-low resilience was significantly higher than depression-low resilience (Bonferroni-corrected *p* < 0.05/4) (Figure 5), while there were no significant differences between the groups with healthy-high resilience and depression-high resilience (*p* > 0.05). However, there were no significant oxy-Hb activation differences among the groups with healthy-low resilience, healthy-high resilience, depression-low resilience and depression-high resilience in negative emotional valence (*p* > 0.05).

We performed a 2 (groups: healthy controls/depression) × 2 (resilience levels: high/low) × 2 (emotional valence: positive/negative) repeated-measures ANOVA for oxy-Hb activation in the LOFC. There were no significant main effects of emotional valence (*p* > 0.05), groups (*p* > 0.05) and resilience level (*p* > 0.05). There was also no significant interaction effect of groups × emotional valence (*p* > 0.05), emotional valence × resilience level (*p* > 0.05), groups × resilience level (*p* > 0.05), or groups × resilience level × emotional valence.

### 3.3 Moderated mediation models

**Figure 6:**
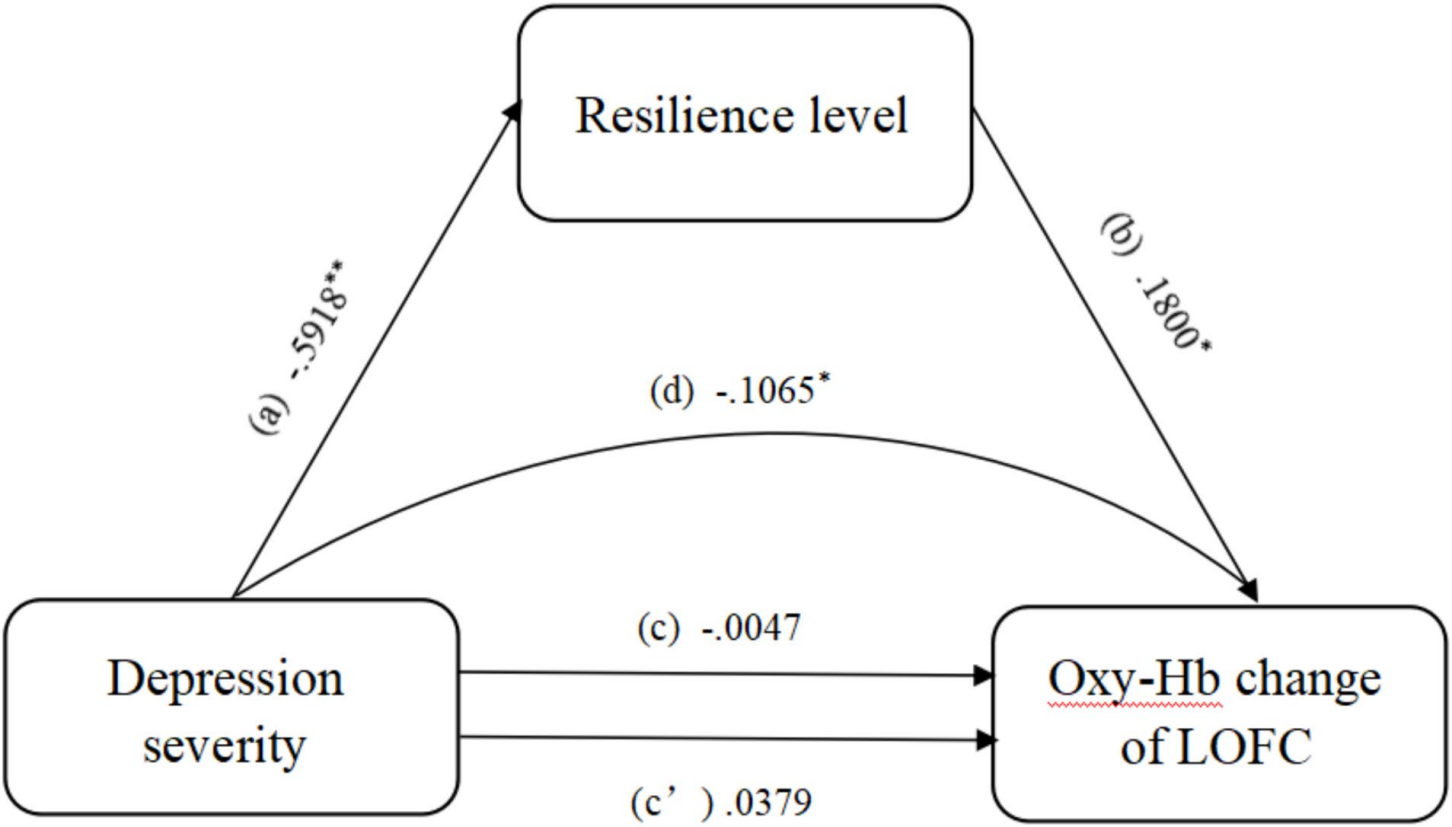
Mediation analysis showing the indirect effect of resilience level on the relationship between depression severity and the change in oxy-hemoglobin in the LOFC. ^*^, 95% CI significant; ^**^, 99.9% CI significant; Oxy-Hb, oxy-hemoglobin; LOFC, left orbitofrontal cortex.

Indirect effect analysis showed that resilience mediated the effects of depression severity on oxy-Hb changes in the LOFC during AMT with negative emotional valence. However, the direct effect analysis showed that depression severity had no effect on the changes in oxy-Hb in the LOFC during AMT on negative emotional valence (Table 2). In other words, the effect of depression on the oxy-Hb changes in the LOFC was completely mediated by the resilience level (Hayes, 2009) (Figure 6).

**Table 2:** Bootstrapping results of the mediation model examine the effects of resilience level on the relationship between the severity of depression and the change in oxy-hemoglobin LOFC (*N* = 237).

	*B*	SE	*	**
			LLCI	ULCI
Total effect of severity of depression on change in oxy-hemoglobin in the LOFC (c)	−0.0047	0.0253	−0.0546	0.0451
Direct effect of the severity of depression on the change in oxy-hemoglobin in the LOFC (c’)	0.0379	0.0311	−0.0234	0.0992
Indirect effect(s) of depression severity on oxy-hemoglobin change in the LOFC (d)	−0.1065	0.0400	−0.1928	−0.0344

B, unstandardized coefficients; SE, standard error. ^*^LLCI: lower limit within the 95% confidence interval of the indirect boot effect. ^**^ULCI: upper limit within the 95% confidence interval of the boot indirect effect. LOFC, left orbitofrontal cortex.

## 4 Discussion

For the first time, this study used fNIRS to investigate differences in oxy-Hb activation among the groups with healthy-low resilience, healthy-high resilience, depression-low resilience and depression-high resilience, and further explored the mediating role of resilience levels in the degree of depression in oxy-Hb activation, to reveal how resilience might protect individuals from depressive symptoms and what neural mechanisms could underlie this “protective” effect. The results showed the following: (1) The oxy-Hb activation of the left DLPFC for the groups with depression-high resilience and healthy-low resilience was significantly higher under the positive emotional valence condition than under the negative emotional valence condition, while the oxy-Hb activation in the left DLPFC was not significantly different between the two emotional valence conditions for the groups with depression-low resilience and healthy-high resilience. (2) Under the condition of positive emotional valence, oxy-Hb activation in the left DLPFC the groups with healthy-low resilience was significantly higher than healthy-high resilience and depression-low resilience. (3) Under the negative emotional valence condition, the resilience level mediated the indirect effect of the degree of depression on the activation of oxy-Hb in the LOFC. This result reveals the neurophysiological basis of brain activation between patients with depression and healthy individuals with different levels of resilience, and the different mechanisms of resilience under different emotional valence conditions under the influence of the degree of depression on brain activation. This study provides a new perspective on interventions and treatments for depression. At the same time, it further improves the accuracy and precision of fNIRS in detecting depression.

### 4.1 Comparison of the hemodynamic difference in the left DLPFC in response to positive and negative valence

In this study, it was found that the oxy-Hb activation of left DLPFC in the depression-high resilience group was significantly higher in the positive emotional valence condition than in the negative emotional valence condition, while there was no significant difference in the oxy-Hb activation of left DLPFC in the depression-low resilience group under positive and negative emotional valence conditions. AM is the long-term memory of personal experiences and is a core part of the self-memory system (Conway and Pleydell-Pearce, 2000). Explicit long-term memory bias in patients with depression is a manifestation of cognitive bias (Everaert et al., 2012). Meanwhile, patients have a recall bias for negative information (Mathews and MacLeod, 2005), and this bias effect is stronger when recalling negative information related to the self (Wisco, 2009). In contrast, patients with depression do not have a positive memory bias for positive events, similar to healthy people (Harmer et al., 2009). Furthermore, fNIRS research has revealed that anxious depression is a more severe subtype than non-anxious depression (Wu et al., 2022; Wu et al., 2024). Wu et al. (2024) used AMT and fNIRS technology to explore differences in oxy-Hb activation in anxious depression, non-anxious depression, and healthy groups under different emotional valence conditions and found that there were no significant differences between positive and negative emotional valence in the anxious depression group. Oxy-Hb activation in the left DLPFC in healthy controls and patients with non-anxious depression was significantly higher in positive emotional valence than in negative emotional valence. This result indicates that patients with anxious depression are deficient in positive emotional valence conditions, suggesting that due to deepening of depression, patients with anxious depression have insufficient oxy-Hb activation in the left DLPFC, which affects the oxy-Hb activation of positive emotional valence, especially processed in this region. Therefore, in this study, the difference in oxy-Hb activation in the left DLPFC between the groups with depression-low resilience and depression-high resilience under the two emotional valence conditions indicated that depressed patients with low resilience may have a more severe depression subtype than those with high resilience, and the processing of positive emotional valence is affected by insufficient oxy-Hb activation in the left DLPFC due to deepening of depression for the group with depression-low resilience. It revealed that the resilience level of depressed patients has a protective effect on the generation and development of depression.

The DLFPC has been associated with cognitive flexibility, reassessment, and impulse control (Steinbeis et al., 2012), and skills such as restructuring and cognitive flexibility have been identified as part of resilience (Iacoviello and Charney, 2014). The left DLPFC has been shown to be a possible neurophysiological mechanism of resilience (Brosch et al., 2022). Many studies have confirmed insufficient activation of the left DLPFC in depressed patients (Shajahan et al., 2002; Zhong et al., 2011; Mannie et al., 2011) and that the degree of activation of this region is negatively correlated with the degree of depression in patients (Shajahan et al., 2002; Speer et al., 2009; Zhong et al., 2011). It is worth proposing that the DLPFC is associated not only with cognitive processes but also with emotional processes. Repeated transcranial magnetic stimulation (rTMS) of the left DLFPC in patients with major depression can increase their empathetic well-being and reduce anhedonia (Light et al., 2019). Resilience is associated with motion regulation, attention, a positive versus negative outlook, reward and motivation, sensitivity to context, response to fear, learning and memory, adaptive social behaviors, and speed of recovery from stress (Southwick and Charney, 2012). Resilient individuals tend to experience fewer depressive symptoms (Fredrickson et al., 2003) and are less likely to develop chronic depression (Bonanno et al., 2004). Research on depression has shown that patients with depression tend to ruminate the process of repeatedly focusing on their own negative emotions (Nolen-Hoeksema, 2000), and that this rumination may be partly due to an inability to suppress negative information (Joormann et al., 2005). Studies have shown that when threats do not materialize, highly resilient individuals successfully suppress anticipated emotions to aid recovery. This ability to successfully suppress negative emotions may be related to a reduced tendency to ruminate, making highly resilient individuals less likely to experience a long-lasting depressive mood and reducing their probability of depression occurring (Nolen-Hoeksema, 2000). In this study, it was found that the activation of the left DLPFC in the depression-high resilience group was significantly higher in the positive emotional valence condition than in the negative emotional valence condition. We concluded that the left DLPFC may be a possible neurophysiological mechanism of resilience and that it is associated with cognitive flexibility. Owing to the protective effect of resilience, the depression-high resilience group had higher cognitive flexibility than the group with lower resilience. By successfully suppressing the negative emotions caused by depression, the depression-high resilience group generated more positive emotions. Furthermore, the left DLPFC is negatively correlated with the severity of depression, and because of the effect of elasticity, activation of this brain region plays an important role in alleviating depression in a certain sense. Thus, the depression-high resilience group exhibited a positive memory bias for positive events, similar to the healthy individuals. It further indicating that resilience is a protective factor in patients with depression, and that the left DLPFC is the neurophysiological basis of resilience as a new means of depression intervention and treatment.

In addition, it should be proposed that left DLPFC activation was significantly higher in the healthy-low resilience group under the positive emotional valence condition than in the negative emotional valence condition, while there was no significant difference in the activation of the left DLPFC in the healthy-high resilience under the two valence conditions. Studies have shown that healthy individuals in the absence of mood disorders tend to focus on positive emotional stimuli. However, depressed patients cannot reasonably allocate their attention to happy or neutral stimuli, and their attention is focused on negative emotional stimuli such as sadness (Gotlib et al., 2004); that is, there is an attentional bias toward negative emotional stimuli (Disner et al., 2011). Functional deficits may lead to attention disengagement disorders (cognitive deficits that inhibit attention to negative stimuli) (Schaefer et al., 2006; Eugene et al., 2010; Mathews and MacLeod, 2005). Healthy controls require greater cognitive effort to separate attention from positive stimuli, while patients with depression require greater cognitive effort to separate attention from negative stimuli (Disner et al., 2011). Numerous studies have found that positive emotional valence is dominated by the left DLPFC (Davidson and Irwin, 1999; Murphy et al., 2003; Wager et al., 2003). Therefore, in this study, the healthy-low resilience group showed significantly higher activation of the left DLPFC under positive emotional valence than under negative emotional valence.

Studies have shown that compared to the group with healthy-low resilience, healthy-high resilience perform better in terms of emotional flexibility (Waugh et al., 2008a), that is, the flexible use of emotional resources to precisely match the needs of the situation (Bonanno et al., 2004). Highly resilient individuals can adapt to stressful situations through active inhibitory control of stressors (Hänsel and von Känel, 2008). In these recurring negative situations, the emotional flexibility hypothesis suggests that resilient individuals should exhibit appropriate emotional and physiological responses when negative events occur and appropriate non-emotional responses when negative events do not occur (Waugh et al., 2008b). Individuals with high resilience recover more quickly (Tugade and Fredrickson, 2004) and completely (Waugh et al., 2008a; Waugh et al., 2008b) than those with low resilience. Studies have also found that resilient individuals use positive emotions to recover from stress and find positive meaning in stress. Highly resilient individuals may be able to build effective coping resources in time to help buffer negative emotional life experiences (both psychological and physical) (Tugade and Fredrickson, 2004). Furthermore, this study found that there were no significant activation differences in the left DLPFC between conditions of positive and negative emotional valence for the healthy-high resilience group, indicating that the activation of the left DLPFC in the healthy-high resilience group quickly returned to normal levels after experiencing emotional events, achieving balance in different emotional states. This further suggests that the healthy-high resilience group perform better in emotional flexibility and that the left DLPFC may be a biomarker of emotional flexibility in resilient individuals. It is worth proposing that emotional variability is adaptive because the type and intensity of emotional responses correspond to the needs required for these real and important emotional events. However, emotional variability can be maladaptive if the type and/or intensity of an individual’s emotional responses do not correspond appropriately to the demands of the environment (Waugh et al., 2011). Studies have found that high levels of emotional variability are a hallmark symptom of some mental health problems, such as borderline personality disorder (Trull et al., 2008). There was no significant difference in the activation of the left DLPFC in the healthy-high resilience group under different emotional valence conditions, further indicating that resilience has a promoting effect on individual mental health and can be used as a new perspective to intervene in mental diseases.

### 4.2 The left DLPFC hemodynamic hyperactivation of positive emotional valence in healthy controls with low resilience

This study also found that, compared to the groups with healthy-high resilience and depression-low resilience, the left DLPFC of the group with healthy-low resilience showed a tendency to oxy-hb hyperactivation under the positive emotional valence condition. Many previous studies have found that the oxy-Hb activation status of the left DLPFC in patients with depression is negatively correlated with the severity of depression (Shajahan et al., 2002; Speer et al., 2009; Zhong et al., 2011). The DLPFC plays an important role in memory representation and is a key brain region for executive control (Chai et al., 2018; Demanet et al., 2016; Leon-Dominguez et al., 2015). Patients with depression show decreased responses to positive scenarios (Sloan et al., 1997), decreased neural activity to positive utterances (Canli et al., 2004), and decreased behavioral responses to reward contingencies (Henriques and Davidson, 2000). These findings suggest that patients with depression have positive emotional information processing deficits and cannot focus excessive attention and cognitive resources on positive information (Levens and Gotlib, 2009). Therefore, under the same low-resilience conditions, depressed patients are insensitive to positive information compared to healthy individuals, and the left DLPFC in the depressed group with low resilience is hypoactivated in the positive emotional state. According to the valence lateralization theory, positive emotional valence is dominated by the left prefrontal cortex, while negative emotional valence is dominated by the right prefrontal cortex (Davidson and Irwin, 1999; Murphy et al., 2003; Wager et al., 2003). An fMRI study of healthy individuals showed that the left DLPFC was activated when participants anticipated positive emotions (Ueda et al., 2003). fMRI studies in patients with depression have found that the lower the left DLPFC signal strength in patients with MDD, the lower the depressed patient’s positive emotional rating, which is consistent not only with the observed lack of left DLPFC activity, but also the inability of patients with MDD to see things positively, even if it is truly positive (Grimm et al., 2008). fNIRS research further revealed that decreased left DLPFC function in patients with depression is associated with the prediction of positive emotions, and abnormal right prefrontal cortex function is associated with the prediction of negative emotions (Akiyama et al., 2018). This evidence further supports the finding that oxy-Hb activation of the left DLPFC in the group with healthy-low resilience was significantly higher than that depression-low resilience under positive emotional valence conditions.

This study also found that activation of the left DLPFC in the group with healthy-low resilience was significantly higher than healthy-low resilience under positive emotional valence conditions. Resilience is characterized by the ability to recover from negative emotional experiences and adapt flexibly to changes in stressful experiences (Block and Block, 1980; Block and Kremen, 1996; Lazarus, 1993). Resilient individuals have an optimistic, enthusiastic, and energetic lifestyle, are curious and open to new experiences, and are characterized by highly positive emotions (Block and Kremen, 1996; Klohnen, 1996). Other evidence suggests that highly resilient individuals use humor (Werner and Smith, 1992), relaxation techniques (Demos, 1989; Wolin and Wolin, 1993), and optimistic thinking (Kumpfer, 1999). Positive emotions are an important component of mental resilience. Many previous studies have found that activation of the left DLPFC is related to active information processing (Ueda et al., 2003; Grimm et al., 2008; Akiyama et al., 2018). In the absence of mood disorders, healthy individuals tend to focus on positive emotional stimuli (Gotlib et al., 2004). The healthy-low resilience group need to overactivate the left DLPFC to maintain more positive emotions and thus maintain mental health, while the healthy-high resilience group are influenced by positive emotions and emotional flexibility and do not need to activate the left DLPFC to provide more positive emotions and maintain mental health.

### 4.3 The level of resilience mediated the indirect effects of depression severity on the hemodynamic changes in the LOFC

Evidence obtained by neuroimaging, neuropathology, and lesion analysis techniques suggests that OFC is involved in the pathophysiology of major depression (Drevets, 2007). Some studies suggest that the severity of depression is negatively correlated with the activation of OFC (Neumeister et al., 2004; Bremner et al., 1997), and some researchers believe that the severity of depression is negatively correlated with the activation of this region (Schneider et al., 1995; Drevets, 1995; Rauch et al., 1994); some studies even believe that there is no relationship between them (Drevets, 2000; Mayberg et al., 1997; Mayberg et al., 1994). However, these studies did not consider the emotional valence of the stimuli. In this study, it was found that under the condition of negative emotional valence, the resilience level played a completely mediated role in the influence of depression severity course on oxy-Hb activation in the LOFC, echoing the conclusion of previous studies on the inconsistent relationship between depression severity and OFC activation, indicating that under the condition of negative emotional valence, the level of resilience is related to the effect of the degree of depression on hemodynamic changes in the LOFC. Studies have shown that the severity of depression is negatively correlated with the level of resilience, which is consistent with previous studies showing that individuals with high resilience are less likely to suffer from depression (Klasen et al., 2015; Ong et al., 2006). This indicates that individuals with severe depressive symptoms may have low psychological resilience and a new approach to depression intervention can be found from the perspective of psychological resilience.

In addition, previous studies have found that the OFC participates in trait resilience through emotional flexibility (Waugh et al., 2008a; Waugh et al., 2008b) and is involved in the expectation of negative outcomes (O'Doherty et al., 2001). Individuals with high resilience show reduced OFC activity in this situation compared to individuals with low resilience because they have greater emotional flexibility (Waugh et al., 2008a; Waugh et al., 2008b). In this study, we found that under the condition of negative emotional valence, the resilience level was positively correlated with the activation of the LOFC hemodynamics, indicating that improving the resilience of individuals during the processing of negative emotional valence may further activate the area of the brain representing emotional flexibility (LOFC). Many previous studies have found that depressed patients pay too much attention to negative emotions and that alleviating depressive symptoms can begin by improving depressive emotions in depressed individuals (Gotlib et al., 2004; Disner et al., 2011; Eugene et al., 2010; Mathews and MacLeod, 2005). In this study, we found that resilience plays a protective role in the influence of the degree of depression on the activation of the LOFC hemodynamic changes when processing negative information, further suggesting that a new approach to the diagnosis and treatment of depression can be developed from the perspective of resilience.

## 5 Limitations

This study has several limitations. First, we did not record the number of events recalled by the different groups with different emotional valences. This may have impacted the research results, and future studies should consider this variable further. Second, HADS was used to assess depression and anxiety scores in all groups. However, the main psychiatric diagnoses evaluate symptoms of anxiety and depression using the HAM-D (Hamilton, 1960; Delaparte et al., 2017; Liu et al., 2015). Therefore, to ensure the reliability of the results of this study, they should be used for further verification. Third, we did not collect information on drug use or the onset time. Therefore, it will be necessary to include these factors in future studies. Fourth, Brosch et al. (2022) showed that using the Connor-Davidson Resilience Scale (CD-RISC) to assess resilience has two shortcomings: first, self-reported resilience does not necessarily portray objective resilience; and second, without assessing adversity, the questionnaires cannot represent the entire process-like concept of resilience. In the future, a more comprehensive and objective approach should be adopted to evaluate resilience and validate the results of this study. Finally, although some interesting conclusions were found in this study, in the group with healthy-high resilience and healthy-low resilience and the group with depression-high resilience and depression-low resilience, the left DLPFC showed an opposite trend in the activated difference between positive emotion valence and negative emotion valence. Our research also explained this difference. However, the stability of this study’s results needs to be validated in different groups.

## 6 Conclusion

In this study, we found that the hemodynamic activation level of the left DLPFC could be used as a biomarker for healthy and depressed individuals with different levels of resilience in different emotional states, suggesting that fNIRS may be a useful tool for diagnosing and characterizing patients with depression with high or low resilience. The level of resilience plays a completely mediated role in the effect of depression severity on hemodynamic changes in the LOFC, suggesting that improving individual resilience may be a new perspective for the diagnosis and intervention of depression.

## Data Availability

The raw data supporting the conclusions of this article will be made available by the authors, without undue reservation.

## References

[ref1] Akiyama T. Koeda M. Okubo Y. Kimura M. (2018). Hypofunction of left dorsolateral prefrontal cortex in depression during verbal fluency task: a multi-channel nearinfrared spectroscopy study. J. Affect. Disord. 231, 83–90. doi: 10.1016/j.jad.2018.01.010, PMID: 29455100

[ref2] Arun K. M. Smitha K. A. Sylaja P. N. Kesavadas C. (2020). Identifying resting-state functional connectivity changes in the motor cortex using fNIRS during recovery from stroke. Brain Topogr. 33, 710–719. doi: 10.1007/s10548-020-00785-2, PMID: 32685998

[ref9001] American Psychological Association, (2013). Diagnostic and Statistical Manual of Mental Disorders, 5th ed. Washington, DC: American Psychiatric Publishing.

[ref3] Bai L. Ma H. Huang Y. X. Luo Y. J. (2005). The development of native Chinese affective picture system-a pretest in 46 college students. Chin. Ment. Health J. 19, 719–722.

[ref5] Berton O. Nestler E. J. (2006). New approaches to antidepressant drug discovery: beyond monoamines. Nat. Rev. Neurosci. 7, 137–151. doi: 10.1038/nrn1846, PMID: 16429123

[ref6] Bjelland I. Dahl A. A. Haug T. T. Neckelmann D. (2002). The validity of the hospital anxiety and depression scale: an updated literature review. J. Psychosom. Res. 52, 77. doi: 10.1016/s0022-3999(01)00296-3 11832252

[ref7] Block J. Block J. H. (1980). “The role of ego-control and ego-resiliencyin the origination of behavior” in The Minnesota symposia on child psychology. ed. Collings W. A., vol. 13 (Hillsdale, NJ: Erlbaum), 39–101.

[ref8] Block J. Kremen A. M. (1996). IQ and ego-resiliency: conceptual and empirical connections and separateness. J. Pers. Soc. Psychol. 70, 349–361. doi: 10.1037/0022-3514.70.2.349, PMID: 8636887

[ref9] Bonanno G. A. Papa A. Lalande K. Westphal M. Coifman K. (2004). The importance of being flexible the ability to both enhance and suppress emotional expression predicts long-term adjustment. Psychol. Sci. 15, 482–487. doi: 10.1111/j.0956-7976.2004.00705.x, PMID: 15200633

[ref10] Bremner J. D. Innis R. B. Salomon R. M. Staib L. H. Ng C. K. Miller H. L.. (1997). Positron emission tomography measurement of cerebral metabolic correlates of tryptophan depletion—induced depressive relapse. Arch. Gen. Psychiatry 54, 364–374. doi: 10.1001/archpsyc.1997.01830160092012, PMID: 9107153

[ref11] Brigadoi S. Ceccherini L. Cutini S. Scarpa F. Scatturin P. Selb J.. (2014). Motion artifacts in functional near-infrared spectroscopy: a comparison of motion correction techniques applied to real cognitive data. NeuroImage 85, 181–191. doi: 10.1016/j.neuroimage.2013.04.082 23639260 PMC3762942

[ref12] Brosch K. Stein F. Meller T. Schmitt S. Yuksel D. Ringwald K. G.. (2022). DLPFC volume is a neural correlate of resilience in healthy high-risk individuals with both childhood maltreatment and familial risk for depression. Psychol. Med. 52, 4139–4145. doi: 10.1017/S0033291721001094, PMID: 33858550 PMC9811272

[ref14] Campbell-Sills L. Cohan S. L. Stein M. B. (2006). Relationship of resilience to personality, coping, and psychiatric symptoms in young adults. Behav. Res. Ther. 44, 585–599. doi: 10.1016/j.brat.2005.05.001, PMID: 15998508

[ref15] Canli T. Sivers H. Thomason M. Whitfield-Gabrieli S. Gabrieli J. Gotlib I. (2004). Brain activation to emotional words in depressed versus healthy subjects. Neuroreport 15, 2585–2588. doi: 10.1097/00001756-200412030-00005 15570157

[ref16] Chai W. J. Hamid A. I. A. Abdullah J. M. (2018). Working memory from the psychological and neurosciences perspectives: a review. Front. Psychol. 9:401. doi: 10.3389/fpsyg.2018.00401 29636715 PMC5881171

[ref17] Connor K. M. Davidson J. R. T. (2003). Development of a new resilience scale: the Connor-Davidson Resilience scale (CD-RISC). Depress. Anxiety 18, 76–82. doi: 10.1002/da.10113, PMID: 12964174

[ref18] Conway M. A. Pleydell-Pearce C. W. (2000). The construction of autobiographical memories in the self-memory system. Psychol. Rev. 107, 261–288. doi: 10.1037/0033-295X.107.2.261, PMID: 10789197

[ref19] Davidson R. J. Irwin W. (1999). The functional neuroanatomy of emotion and affective style. Trends Cogn. Sci. 3, 11–21. doi: 10.1016/S1364-6613(98)01265-0, PMID: 10234222

[ref20] Delaparte L. Yeh F. C. Adams P. Malchow A. Trivedi M. H. Oquendo M. A.. (2017). A comparison of structural connectivity in anxious depression versus nonanxious depression. J. Psychiatr. Res. 89, 38–47. doi: 10.1016/j.jpsychires.2017.01.012, PMID: 28157545 PMC5374003

[ref21] Demanet J. Liefooghe B. Hartstra E. Wenke D. De Houwer J. Brass M. (2016). There is more into “doing” than “knowing”: the function of the right inferior frontal sulcus is specific for implementing versus memorising verbal instructions. NeuroImage 141, 350–356. doi: 10.1016/j.neuroimage.2016.07.059, PMID: 27480625

[ref22] Demos V. (1989). Maintenance and loss of traditional gender boundaries in two Greek orthodox communities. J. Hellenic Diaspora. Development 16, 77–93.

[ref23] Disner S. G. Beevers C. G. Haigh E. A. P. Beck A. T. (2011). Neural mechanisms of the cognitive model of depression. Nat. Rev. Neurosci. 12, 467–477. doi: 10.1038/nrn3027, PMID: 21731066

[ref24] Donnellan M. B. Conger K. J. McAdams K. K. Neppl T. K. (2009). Personal characteristics and resilience to economic hardship and its consequences: conceptual issues and empirical illustrations. J. Pers. 77, 1645–1676. doi: 10.1111/j.1467-6494.2009.00596.x, PMID: 19796065 PMC2787744

[ref25] Drevets W. C. (1995). Regional blood flow changes in response to phobic anxiety and habituation. J. Cereb. Blood Flow Metab. 15:S856.

[ref26] Drevets W. C. (2000). Neuroimaging studies of mood disorders. Biol. Psychiatry 48, 813–829. doi: 10.1016/S0006-3223(00)01020-9, PMID: 11063977

[ref27] Drevets W. C. (2007). Orbitofrontal cortex function and structure in depression. Ann. N. Y. Acad. Sci. 1121, 499–527. doi: 10.1196/annals.1401.029 17872395

[ref28] Eugene F. Joormann J. Cooney R. E. Atlas L. Y. Gotlib I. H. (2010). Neural correlates of inhibitory deficits in depression. Psychiatry Res. 181, 30–35. doi: 10.1016/j.pscychresns.2009.07.010, PMID: 19962859 PMC2795107

[ref29] Everaert J. Koster E. H. Derakshan N. (2012). The combined cognitive bias hypothesis in depression. Clin. Psychol. Rev. 32, 413–424. doi: 10.1016/j.cpr.2012.04.003, PMID: 22681914

[ref30] Ferrari A. J. Charlson F. J. Norman R. E. Flaxman A. D. Patten S. B. Vos T.. (2013). The epidemiological modelling of major depressive disorder: application for the global burden of disease study 2010. PLoS One 8:e69637. doi: 10.1371/journal.pone.0069637, PMID: 23922765 PMC3726670

[ref31] Fredrickson B. L. Tugade M. M. Waugh C. E. Larkin G. R. (2003). What good are positive emotions in crisis? A prospective study of resilience and emotions following the terrorist attacks on the United States on September 11th, 2001. J. Pers. Soc. Psychol. 84, 365–376. doi: 10.1037/0022-3514.84.2.365, PMID: 12585810 PMC2755263

[ref32] Friedman A. K. Juarez B. Ku S. M. Zhang H. Calizo R. C. Walsh J. J.. (2016). KCNQ channel openers reverse depressive symptoms via an active resilience mechanism. Nat. Commun. 7:11671. doi: 10.1038/ncomms11671 27216573 PMC4890180

[ref33] Friedman A. K. Walsh J. J. Juarez B. Ku S. M. Chaudhury D. Wang J.. (2014). Enhancing depression mechanisms in midbrain dopamine neurons achieves homeostatic resilience. Science 344, 313–319. doi: 10.1126/science.1249240, PMID: 24744379 PMC4334447

[ref34] Garmezy N. (1985). Competence and adaptation in adult schizophrenic patients and children at risk. In Research in the Schizophrenic Disorders: The Stanley R. Dean Award Lectures. Vol. II (pp. 69-112) Dordrecht: Springer Netherlands.

[ref35] Gotlib I. H. Krasnoperova E. Yue D. N. Joormann J. (2004). Attentional biases for negative interpersonal stimuli in clinical depression. J. Abnorm. Psychol. 113, 127–135. doi: 10.1037/0021-843X.113.1.121 14992665

[ref36] Grimm S. Beck J. Schuepbach D. Hell D. Boesiger P. Bermpohl F.. (2008). Imbalance between left and right dorsolateral prefrontal cortex in major depression is linked to negative emotional judgment: an fMRI study in severe major depressive disorder. Biol. Psychiatry 63, 369–376. doi: 10.1016/j.biopsych.2007.05.033, PMID: 17888408

[ref37] Hamilton M. (1960). A rating scale for depression. J. Neurol. Neurosurg. Psychiatry 23, 56–62. doi: 10.1136/jnnp.23.1.56, PMID: 14399272 PMC495331

[ref38] Han M. H. Nestler E. J. (2017). Neural substrates of depression and resilience. Neurotherapeutics 14, 677–686. doi: 10.1007/s13311-017-0527-x, PMID: 28397115 PMC5509625

[ref39] Hänsel A. von Känel R. (2008). The ventro-medial prefrontal cortex: a major link between the autonomic nervous system, regulation of emotion, and stress reactivity? BioPsychoSocial Med. 2, 1–5. doi: 10.1186/1751-0759-2-21, PMID: 18986513 PMC2590602

[ref40] Harmer C. J. Goodwin G. M. Cowen P. J. (2009). Why do antidepressants take so long to work? A cognitive neuropsychological model of antidepressant drug action. Br. J. Psychiatry 195, 102–108. doi: 10.1192/bjp.bp.108.051193, PMID: 19648538

[ref41] Hayes A. F. (2009). Beyond baron and Kenny: statistical mediation analysis in the new millennium. Commun. Monogr. 76, 408–420. doi: 10.1080/03637750903310360

[ref42] Henriques J. B. Davidson R. J. (2000). Decreased responsiveness to reward in depression. Cogn. Emot. 14, 711–724. doi: 10.1080/02699930050117684

[ref43] Herrero M. J. Blanch J. Peri J. M. Pablo J. D. Bulbena A. (2003). A validation study of the hospital anxiety and depression scale (HADS) in a Spanish population. Gen. Hosp. Psychiatry 25, 277–283. doi: 10.1016/S0163-8343(03)00043-4, PMID: 12850660

[ref44] Ho C. S. H. Lim L. J. H. Lim A. Q. Chan N. H. C. Ho R. C. M. (2020). Diagnostic and predictive applications of functional near-infrared spectroscopy for major depressive disorder: a systematic review. Front. Psych. 11:378. doi: 10.3389/fpsyt.2020.00378, PMID: 32477179 PMC7232562

[ref45] Huppert T. J. Franceschini M. A. Diamond S. G. Boas D. A. (2009). HomER: a review of time-series analysis methods for near-infrared spectroscopy of the brain. Appl. Opt. 48, 280–298. doi: 10.1364/AO.48.00D280 PMC2761652 19340120

[ref46] Huppert T. J. Hoge R. D. Diamond S. G. Franceschini M. A. Boas D. A. (2006). A temporal comparison of BOLD, ASL, and NIRS hemodynamic responses to motor stimuli in adult humans. NeuroImage 29, 368–382. doi: 10.1016/j.neuroimage.2005.08.065, PMID: 16303317 PMC2692693

[ref47] Iacoviello B. M. Charney D. S. (2014). Psychosocial facets of resilience: implications for preventing posttrauma psychopathology, treating trauma survivors, and enhancing community resilience. Eur. J. Psychotraumatol. 5:23970. doi: 10.3402/ejpt.v5.23970, PMID: 25317258 PMC4185137

[ref48] Jaccard J. Turrisi R. (2003). Interaction effects in multiple regression: Sage Publications Sage university papers series. Quantitative applications in the social sciences, London: Sage Publications. Vol. no. 07-072.

[ref49] Joormann J. Hertel P. T. Brozovich F. Gotlib I. H. (2005). Remembering the good, forgetting the bad: intentional forgetting of emotional material in depression. J. Abnorm. Psychol. 114, 640–648. doi: 10.1037/0021-843X.114.4.640, PMID: 16351371

[ref51] Kessler R. C. McGonagle K. A. Zhao S. Nelson C. B. Hughes M. Eshleman S.. (1994). Lifetime and 12-month prevalence of DSM-III-R psychiatric disorders in the United States: results from the National Comorbidity Survey. Arch. Gen. Psychiatry 51, 8–19. doi: 10.1001/archpsyc.1994.03950010008002 8279933

[ref52] Klasen F. Otto C. Kriston L. Patalay P. Schlack R. Ravens-Sieberer U. (2015). Risk and protective factors for the development of depressive symptoms in children and adolescents: results of the longitudinal BELLA study. Eur. Child Adolesc. Psychiatry 24, 695–703. doi: 10.1007/s00787-014-0637-5, PMID: 25432629

[ref53] Klohnen E. C. (1996). Conceptual analysis and measurement of the construct of ego-resiliency. J. Pers. Soc. Psychol. 70, 1067–1079. doi: 10.1037/0022-3514.70.5.1067, PMID: 8656335

[ref54] Kumpfer K. L. (1999). Outcome measures of interventions in the study of children of substance-abusing parents. Pediatrics 103:1128. doi: 10.1542/peds.103.S2.1128 10224200

[ref55] Lai C. Y. Y. Ho C. S. H. Lim C. R. Ho R. C. M. (2017). Functional near-infrared spectroscopy in psychiatry. BJPsych Adv. 23, 324–330. doi: 10.1192/apt.bp.115.015610

[ref56] Lazarus R. S. (1993). From psychological stress to the emotions: a history of changing outlooks. Annu. Rev. Psychol. 44, 1–22. doi: 10.1146/annurev.ps.44.020193.000245, PMID: 8434890

[ref57] Leon-Dominguez U. Martín-Rodríguez J. F. León-Carrión J. (2015). Executive n-back tasks for the neuropsychological assessment of working memory. Behav. Brain Res. 292, 167–173. doi: 10.1016/j.bbr.2015.06.002 26068585

[ref58] Leve L. D. Fisher P. A. Chamberlain P. (2009). Multidimensional treatment foster care as a preventive intervention to promote resiliency among youth in the child welfare system. J. Pers. 77, 1869–1902. doi: 10.1111/j.1467-6494.2009.00603.x, PMID: 19807861 PMC2787781

[ref59] Levens S. M. Gotlib I. H. (2009). Impaired selection of relevant positive information in depression. Depress. Anxiety 26, 403–410. doi: 10.1002/da.20565, PMID: 19347861 PMC2836936

[ref60] Lieberman M. D. Eisenberger N. I. Crockett M. J. Tom S. M. Pfeifer J. H. Way B. M. (2007). Putting feelings into words: affect labeling disrupts amygdala activity in response to affective stimuli. Psychol. Sci. 18, 421–428. doi: 10.1111/j.1467-9280.2007.01916.x, PMID: 17576282

[ref61] Light S. N. Bieliauskas L. A. Taylor S. F. (2019). Measuring change in anhedonia using the ‘happy faces’ task pre- to post-repetitive transcranial magnetic stimulation (rTMS) treatment to left dorsolateral prefrontal cortex in major depressive disorder (MDD): relation to empathic happiness. Transl. Psychiatry 9, 1–10. doi: 10.1038/s41398-019-0549-8, PMID: 31481688 PMC6722063

[ref62] Liu C. H. Ma X. Song L. P. Fan J. Wang W. D. Lv X. Y.. (2015). Abnormal spontaneous neural activity in the anterior insular and anterior cingulate cortices in anxious depression. Behav. Brain Res. 281, 339–347. doi: 10.1016/j.bbr.2014.11.047, PMID: 25513974

[ref63] Liu X. Sun G. Zhang X. Xu B. Shen C. Shi L.. (2014). Relationship between the prefrontal function and the severity of the emotional symptoms during a verbal fluency task in patients with major depressive disorder: a multi-channel NIRS study. Prog. Neuro-Psychopharmacol. Biol. Psychiatry 54, 114–121. doi: 10.1016/j.pnpbp.2014.05.005, PMID: 24842802

[ref65] Luthar S. S. Cicchetti D. Becker B. (2000). The construct of resilience: a critical evaluation and guidelines for future work. Child Dev. 71, 543–562. doi: 10.1111/1467-8624.00164, PMID: 10953923 PMC1885202

[ref68] Mannie Z. N. Taylor M. J. Harmer C. J. Cowen P. J. Norbury R. (2011). Frontolimbic responses to emotional faces in young people at familial risk of depression. J. Affect. Disord. 130, 127–132. doi: 10.1016/j.jad.2010.09.030, PMID: 20952073

[ref69] Masten A. S. (2014a). Global perspectives on resilience in children and youth. Child Dev. 85, 6–20. doi: 10.1111/cdev.12205, PMID: 24341286

[ref70] Masten A. S. (2014b). Invited commentary: Resilience and positive youth development frameworks in developmental science. J. Youth Adolesc. 43, 1018–1024. doi: 10.1007/s10964-014-0118-7 24723048

[ref71] Mathews A. MacLeod C. (2005). Cognitive vulnerability to emotional disorders. Annu. Rev. Clin. Psychol. 1, 167–195. doi: 10.1146/annurev.clinpsy.1.102803.143916 17716086

[ref72] Mayberg H. S. Brannan S. K. Mahurin R. K. Jerabek P. A. Brickman J. S. Tekell J. L.. (1997). Cingulate function in depression: a potential predictor of treatment response. Neuroreport 8, 1057–1061. doi: 10.1097/00001756-199703030-00048, PMID: 9141092

[ref73] Mayberg H. S. Lewis P. J. Regenold W. Wagner H. N. (1994). Paralimbic hypoperfusion in unipolar depression. J. Nucl. Med. 35, 929–934. doi: 10.1002/jcu.1870220513 8195877

[ref76] Murphy F. C. Nimmo-Smith I. Lawrence A. D. (2003). Functional neuroanatomy of emotions: a meta-analysis. Cogn. Affect. Behav. Neurosci. 3, 207–233. doi: 10.3758/CABN.3.3.207, PMID: 14672157

[ref77] Nestler E. J. Barrot M. DiLeone R. J. Eisch A. J. Gold S. J. Monteggia L. M. (2002). Neurobiology of depression. Neuron 34, 13–25. doi: 10.1016/S0896-6273(02)00653-0 11931738

[ref78] Nestler E. J. Carlezon W. A. (2006). The mesolimbic dopamine reward circuit in depression. Biol. Psychiatry 59, 1151–1159. doi: 10.1016/j.biopsych.2005.09.018, PMID: 16566899

[ref79] Neumeister A. Nugent A. C. Waldeck T. Geraci M. Schwarz M. Bonne O.. (2004). Neural and behavioral responses to tryptophan depletion in unmedicatedpatients with remitted major depressive disorder and controls. Arch. Gen. Psychiatry 61, 765–773. doi: 10.1001/archpsyc.61.8.765, PMID: 15289275

[ref80] Nolen-Hoeksema S. (2000). The role of rumination in depressive disorders and mixed anxiety/depressive symptoms. J. Abnorm. Psychol. 109, 504–511. doi: 10.1037/0021-843X.109.3.504, PMID: 11016119

[ref81] O'Doherty J. Kringelbach M. L. Rolls E. T. Hornak J. Andrews C. (2001). Abstract reward and punishment representations in the human orbitofrontal cortex. Nat. Neurosci. 4, 95–102. doi: 10.1038/82959, PMID: 11135651

[ref9002] Okamoto M. Dan H. Sakamoto K. Takeo K. Shimizu K. Kohno S. (2004). Three-dimensional probabilistic anatomical cranio-cerebral correlation via the international 10–20 system oriented for transcranial functional brain map**. Neuroimage. 21:99–111. doi: 10.1016/j.neuroimage.2003.08.026 14741647

[ref82] Ong A. D. Bergeman C. S. Bisconti T. L. Wallace K. A. (2006). Psychological resilience, positive emotions, and successful adaptation to stress in later life. J. Pers. Soc. Psychol. 91, 730–749. doi: 10.1037/0022-3514.91.4.730, PMID: 17014296

[ref83] Ong A. D. Bergeman C. S. Boker S. M. (2009). Resilience comes of age: defining features in later adulthood. J. Pers. 77, 1777–1804. doi: 10.1111/j.1467-6494.2009.00600.x, PMID: 19807864 PMC2807734

[ref84] Ong S. K. Husain S. F. Wee H. N. Ching J. Kovalik J. P. Cheng M. S.. (2021). Integration of the cortical haemodynamic response measured by functional near-infrared spectroscopy and amino acid analysis to aid in the diagnosis of major depressive disorder. Diagnostics 11:1978. doi: 10.3390/diagnostics11111978, PMID: 34829325 PMC8617819

[ref87] Phillips M. L. Ladouceur C. D. Drevets W. C. (2008). A neural model of voluntary and automatic emotion regulation: implications for understanding the pathophysiology and neurodevelopment of bipolar disorder. Mol. Psychiatry 13, 833–857. doi: 10.1038/mp.2008.65, PMID: 18574483 PMC2745893

[ref88] Pinti P. Tachtsidis I. Hamilton A. Hirsch J. Aichelburg C. Gilbert S.. (2020). The present and future use of functional near- infrared spectroscopy (fNIRS) for cognitive neuroscience. Ann. N. Y. Acad. Sci. 1464, 5–29. doi: 10.1111/nyas.13948, PMID: 30085354 PMC6367070

[ref89] Piper S. K. Krueger A. Koch S. P. Mehnert J. Habermehl C. Steinbrink J.. (2014). A wearable multi-channel fNIRS system for brain imaging in freely moving subjects. NeuroImage 85, 64–71. doi: 10.1016/j.neuroimage.2013.06.062, PMID: 23810973 PMC3859838

[ref90] Preacher K. J. Hayes A. F. (2008). Asymptotic and resampling strategies for assessing and comparing indirect effects in multiple mediator models. Behav. Res. Methods 40, 879–891. doi: 10.3758/BRM.40.3.879 18697684

[ref91] Rauch S. L. Jenike M. A. Alpert N. M. Baer L. Breiter H. C. Savage C. R.. (1994). Regional cerebral blood flow measured during symptom provocation in obsessive-compulsive disorder using oxygen 15—labeled carbon dioxide and positron emission tomography. Arch. Gen. Psychiatry 51, 62–70. doi: 10.1001/archpsyc.1994.03950010062008, PMID: 8279930

[ref93] Reynaud E. Guedj E. Souville M. Trousselard M. Zendjidjian X. El Khoury-Malhame M.. (2013). Relationship between emotional experience and resilience: an fMRI study in fire-fighters. Neuropsychologia 51, 845–849. doi: 10.1016/j.neuropsychologia.2013.01.007, PMID: 23369802

[ref94] Rolls E. T. (1999). Spatial view cells and the representation of place in the primate hippocampus. Hippocampus 9, 467–480. doi: 10.1002/(SICI)1098-1063(1999)9:4<467::AID-HIPO13>3.0.CO;2-F 10495028

[ref95] Russo S. J. Murrough J. W. Han M. H. Charney D. S. Nestler E. J. (2012). Neurobiology of resilience. Nat. Neurosci. 15, 1475–1484. doi: 10.1038/nn.3234, PMID: 23064380 PMC3580862

[ref96] Salehinejad M. A. Nejati V. Derakhshan M. (2017). Neural correlates of trait resiliency: evidence from electrical stimulation of the dorsolateral prefrontal cortex (dLPFC) and orbitofrontal cortex (OFC). Personal. Individ. Differ. 106, 209–216. doi: 10.1016/j.paid.2016.11.005

[ref97] Sanchez-Lopez A. De Raedt R. Puttevils L. Koster E. H. W. Baeken C. Vanderhasselt M. A. (2020). Combined effects of tDCS over the left DLPFC and gaze-contingent training on attention mechanisms of emotion regulation in low-resilient individuals. Prog. Neuro-Psychopharmacol. Biol. Psychiatry 108:110177. doi: 10.1016/j.pnpbp.2020.110177 33189857

[ref98] Schaefer H. S. Putnam K. M. Benca R. M. Davidson R. J. (2006). Event-related functional magnetic resonance imaging measures of neural activity to positive social stimuli in pre- and post-treatment depression. Biol. Psychiatry 60, 974–986. doi: 10.1016/j.biopsych.2006.03.024, PMID: 16780808

[ref99] Schneider F. Gur R. E. Mozley L. H. Smith R. J. Mozley P. D. Censits D. M.. (1995). Mood effects on limbic blood flow correlate with emotional self-rating: a PET study with oxygen-15 labeled water. Psychiatry Res. Neuroimaging 61, 265–283. doi: 10.1016/0925-4927(95)02678-q, PMID: 8748470

[ref100] Scholkmann F. Spichtig S. Muehlemann T. Wolf M. (2010). How to detect and reduce movement artifacts in near-infrared imaging using moving standard deviation and spline interpolation. Physiol. Meas. 31, 649–662. doi: 10.1088/0967-3334/31/5/004, PMID: 20308772

[ref101] Shajahan P. M. Glabus M. F. Steele J. D. Doris A. B. Anderson K. Jenkins J. A.. (2002). Left dorso-lateral repetitive transcranial magnetic stimulation affects cortical excitability and functional connectivity, but does not impair cognition in major depression. Prog. Neuro-Psychopharmacol. Biol. Psychiatry 26, 945–954. doi: 10.1016/s0278-5846(02)00210-5, PMID: 12369271

[ref102] Shrout P. E. Bolger J. (2002). Mediation in experimental and nonexperimental studies: new procedures and recommendations. Psychol. Methods 7:422. doi: 10.1037/1082-989X.7.4.422 12530702

[ref103] Sloan D. M. Strauss M. E. Quirck S. W. Sajatovic M. (1997). Subjective and expressive emotional responses in depression. J. Affect. Disord. 46, 135–141. [PubMed: 9479617]. doi: 10.1016/S0165-0327(97)00097-9 9479617

[ref104] Snaith P. R. (1992). Availability of the hospital anxiety and depression (HAD) scale. Br. J. Psychiatry 161:422. doi: 10.1192/bjp.161.3.422a 1393323

[ref105] Southwick S. M. Charney D. S. (2012). The science of resilience: implications for the prevention and treatment of depression. Science 338, 79–82. doi: 10.1126/science.1222942, PMID: 23042887

[ref106] Speer A. M. Benson B. E. Kimbrell T. K. Wassermann E. M. Willis M. W. Herscovitch P.. (2009). Opposite effects of high and low frequency rTMS on mood in depressed patients: relationship to baseline cerebral activity on PET. J. Affect. Disord. 115, 386–394. doi: 10.1016/j.jad.2008.10.006, PMID: 19027962 PMC2779113

[ref107] Steinbeis N. Bernhardt B. C. Singer T. (2012). Impulse control and underlying functions of the left DLPFC mediate age-related and age-independent individual differences in strategic social behavior. Neuron 73, 1040–1051. doi: 10.1016/j.neuron.2011.12.027, PMID: 22405212

[ref108] Strangman G. Culver J. P. Thompson J. H. Boas D. A. (2002). A quantitative comparison of simultaneous BOLD fMRI and NIRS recordings during functional brain activation. NeuroImage 17, 719–731. doi: 10.1006/nimg.2002.1227, PMID: 12377147

[ref109] Toseeb U. Brage S. Corder K. Dunn V. J. Jones P. B. Owens M.. (2014). Exercise and depressive symptoms in adolescents: a longitudinal cohort study. JAMA Pediatr. 168, 1093–1100. doi: 10.1001/jamapediatrics.2014.1794, PMID: 25317674

[ref110] Trull T. J. Solhan M. B. Tragesser S. L. Jahng S. Wood P. K. Piasecki T. M.. (2008). Affective instability: measuring a core feature of borderline personality disorder with ecological momentary assessment. J. Abnorm. Psychol. 117, 647–661. doi: 10.1037/a0012532, PMID: 18729616

[ref111] Tsujii N. Mikawa W. Akashi H. Tsujimoto E. Adachi T. Kirime E.. (2014). Right temporal activation differs between melancholia and nonmelancholic depression: a multichannel near-infrared spectroscopy study. J. Psychiatr. Res. 55, 1–7. doi: 10.1016/j.jpsychires.2014.04.003, PMID: 24780385

[ref112] Tugade M. M. Fredrickson B. L. (2004). Resilient individuals use positive emotions to bounce back from negative emotional experiences. J. Pers. Soc. Psychol. 86, 320–333. doi: 10.1037/0022-3514.86.2.320, PMID: 14769087 PMC3132556

[ref113] Ueda K. Okamoto Y. Okada G. Yamashita H. Hori T. Yamawaki S. (2003). Brain activity during expectancy of emotional stimuli: an fMRI study. Neuroreport 14, 51–55. doi: 10.1097/00001756-200301200-00010 12544830

[ref114] Wager T. D. Phan K. L. Liberzon I. Taylor S. F. (2003). Valence, gender, and lateralization of functional brain anatomy in emotion: a meta-analysis of findings from neuroimaging. NeuroImage 19, 513–531. doi: 10.1016/S1053-8119(03)00078-8, PMID: 12880784

[ref115] Waugh C. E. Fredrickson B. L. Taylor S. F. (2008b). Adapting to life’s slings and arrows: individual differences in resilience when recovering from an anticipated threat. J. Res. Pers. 42, 1031–1046. doi: 10.1016/j.jrp.2008.02.005, PMID: 19649310 PMC2711547

[ref116] Waugh C. E. Thompson R. J. Gotlib I. H. (2011). Flexible emotional responsiveness in trait resilience. Emotion 11, 1059–1067. doi: 10.1037/a0021786, PMID: 21707168 PMC3183326

[ref117] Waugh C. E. Wager T. D. Fredrickson B. L. Noll D. C. Taylor S. F. (2008a). The neural correlates of trait resilience when anticipating and recovering from threat. Soc. Cogn. Affect. Neurosci. 3, 322–332. doi: 10.1093/scan/nsn024, PMID: 19015078 PMC2607054

[ref118] Werner E. E. Smith R. S. (1992). Overcoming the odds: High risk children from birth to adulthood. Ithaca, NY: Cornell University Press.

[ref119] Whelan-Goodinson R. Ponsford J. Sch¨oNberger M. (2009). Validity of the hospital anxiety and depression scale to assess depression and anxiety following traumatic brain injury as compared with the structured clinical interview for DSM-IV. J. Affect. Disord. 114, 94–102. doi: 10.1016/j.jad.2008.06.007 18656266

[ref120] White D. Leach C. Sims R. Atkinson M. Cottrell D. (1999). Validation of the hospital anxiety and depression scale for use with adolescents. Br. J. Psychiatry 175, 452–454. doi: 10.1192/bjp.175.5.452, PMID: 10789277

[ref121] Wickens T. D. Keppel G. (2004). Design and analysis: A researcher's handbook. Upper Saddle River, NJ: Pearson Prentice-Hall, –958.

[ref124] Wisco B. E. (2009). Depressive cognition: self-reference and depth of processing. Clin. Psychol. Rev. 29, 382–392. doi: 10.1016/j.cpr.2009.03.003, PMID: 19346043

[ref125] Wolin S. J. Wolin S. (1993). The resilient self: How survivors of troubled families rise above adversity. NewYork: Villard.

[ref126] Wu H. Li T. Peng C. Yang C. Bian Y. Li X.. (2022). The right prefrontal cortex (PFC) can distinguish anxious depression from non-anxious depression: a promising functional near infrared spectroscopy study (fNIRS). J. Affect. Disord. 317, 319–328. doi: 10.1016/j.jad.2022.08.024, PMID: 36007594

[ref127] Wu H. Lu B. Xiang N. Qiu M. Da H. Xiao Q.. (2024). Different activation in dorsolateral prefrontal cortex between anxious depression and non-anxious depression during an autobiographical memory task: a fNIRS study. J. Affect. Disord. 362, 585–594. doi: 10.1016/j.jad.2024.07.031, PMID: 39019227

[ref9003] Yi‐Frazier J. P. Smith R. E. Vitaliano P. P. Yi J. C. Mai S. Hillman M.. (2010). A person‐focused analysis of resilience resources and coping in patients with diabetes. Stress and health: Journal of the International Society for the Investigation of Stress, 26, 51–60. doi: 10.1002/smi.1258 PMC2880488 20526415

[ref128] Yu X. Zhang J. (2007). Fact or analysis and psycho metric evaluation of the Connor-Davidson resilience scale (CD-RISC) in Chinese people. Soc. Behav. Personal. 35, 19–30. doi: 10.2224/sbp.2007.35.1.19

[ref129] Zhang H. Dong W. Dang W. Quan W. Tian J. Chen R.. (2015). Near-infrared spectroscopy for examination of prefrontal activation during cognitive tasks in patients with major depressive disorder: a meta-analysis of observational studies. Psychiatry Clin. Neurosci. 69, 22–33. doi: 10.1111/pcn.12209, PMID: 24897940

[ref130] Zhang Y. Li X. Guo Y. Zhang Z. Xu F. Xiang N.. (2022). Dorsolateral prefrontal activation in emotional autobiographical task in depressed and anxious college students: an fNIRS study. Int. J. Environ. Res. Public Health 19:14335. doi: 10.3390/ijerph192114335, PMID: 36361214 PMC9657988

[ref131] Zhang Y. Su L. Da H. Ji B. Ao Q. Shi H. (2025). The role of dorsolateral and orbitofrontal cortex in depressed with insomniacs population: a large-scale fNIRS study. Curr. Psychol., 44:9377–9389. doi: 10.1007/s12144-025-07753-8

[ref132] Zheng X. Da H. Pan X. Bian Y. Li X. Zhang Y. (2023). Dorsolateral prefrontal activation in depressed young adults with and without suicidal ideation during an emotional autobiographical memory task: a fNIRS study. J. Affect. Disord. 326, 216–224. doi: 10.1016/j.jad.2023.01.115 36736791

[ref133] Zhong M. T. Wang X. Xiao J. Yi J. Y. Zhu X. L. Liao J.. (2011). Amygdala hyperactivation and prefrontal hypoactivation in subjects with cognitive vulnerability to depression. Biol. Psychol. 88, 233–242. doi: 10.1016/j.biopsycho.2011.08.007, PMID: 21878364

[ref9004] Zigmond A. S. Snaith R. P., (1983). The hospital anxiety and depression scale. Traduction française: J.F. L’epine. Acta Psychiatr. Scand. 67, 361–370. doi: 10.1111/j.1600-0447.1983.tb09716.x 6880820

